# Invertebrate-Derived DNA (iDNA) to Identify Sand Flies’ Bloodmeal: A Molecular Approach to Identifying Hosts in Blood-Feeding Vectors of Leishmaniasis

**DOI:** 10.3390/microorganisms13122650

**Published:** 2025-11-21

**Authors:** Bruno Oliveira Cova, Bruno Henrique Saranholi, Carla Cristina Gestich, Paulo Roberto Machado, Adriano Figueiredo Monte-Alegre, Albert Schriefer

**Affiliations:** 1 Serviço de Imunologia, Hospital Universitário Professor Edgard Santos, Universidade Federal da Bahia (UFBA), Salvador 40170-110, BA, Brazil; prlmachado@hotmail.com (P.R.M.); adriano.monte@ufba.br (A.F.M.-A.); nab.schriefer@gmail.com (A.S.); 2 Programa de Pós-graduação em Ciências da Saúde, Faculdade de Medicina da Bahia, Universidade Federal da Bahia (UFBA), Salvador 40170-110, BA, Brazil; 3 Instituto Tecnológico Vale, Belém 66055-090, PA, Brazil; bruno.saranholi@gmail.com; 4 Departamento de Genética e Evolução, Universidade Federal de São Carlos, São Carlos 13565-905, SP, Brazil; carlagestich@gmail.com; 5 Departamento de Ciências da Biointeração, Instituto de Ciências da Saúde, Universidade Federal da Bahia (UFBA), Salvador 40170-110, BA, Brazil

**Keywords:** Phlebotominae, bloodmeal, metabarcoding, iDNA, *Leishmania*

## Abstract

DNA metabarcoding data obtained by next generation sequencing (NGS) has been used to identify species in mixed biological samples, such as DNA from the gut content of invertebrates that feed on vertebrates (invertebrate-derived DNA, iDNA). This investigation employed DNA metabarcoding approach to determine vertebrate hosts of female phlebotomine sand flies, blood-feeding leishmaniasis vectors. We evaluated performance across three mitochondrial markers: a mammal-specific mini-barcode (16S rRNA), a pan-vertebrate mini-barcode (12S rRNA), and a standard CytB barcode region. Phlebotomine sand flies collections occurred in the Cacao Region of Southeastern Bahia, Brazil, an American Tegumentary Leishmaniasis (ATL) endemic zone. Our analysis examined iDNA from forty female specimens pooled in thirteen samples of seven sand fly species, including confirmed ATL vectors. Metabarcoding-derived operational taxonomic units (OTUs) underwent taxonomic assignment through comparison with GenBank NCBI^®^ reference databases. Results identified twenty vertebrate OTUs: primates (four OTUs), rodents (four), ungulates (five), marsupials (one), plus a domestic dog and a chicken. Notably, non-mammalian taxa, including reptiles (one OTU) and amphibians (three), were detected. The iDNA metabarcoding approach allowed us to accurately sample the diversity of phlebotomine sandflies’ bloodmeals in a single specimen of a non-engorged female sand fly with mixed feeding.

## 1. Introduction

Vertebrate-vector interactions are fundamental to the transmission and establishment of leishmaniasis endemic foci, yet many aspects of these complex networks remain poorly understood [1]. Investigating the feeding habits of phlebotomine sand flies (Diptera: Psychodidae) provides indirect data regarding the presence of vertebrates that may act as potential reservoirs/hosts of *Leishmania* spp. Ross, 1903 (Euglenozoa: Trypanosomatidae: Leishmaniinae) [2,3]. Over the last two decades, laboratory methods for detecting bloodmeal remnants in engorged phlebotomine sand flies have evolved, underscoring their critical epidemiological significance [4].

The precipitin immunotest and enzyme-linked immunosorbent assay (ELISA) can be employed for the spectrum of antisera, including those for birds, armadillo, chicken, dog, goat, opossum, equine, feline, human, sheep, rodent, and pig, or family-specific antisera. Additionally, in both methodologies, there is a lack of a pattern cut-off point to provide information on the identification of bloodmeal sources for sand fly species, other than the risk of bias due to the cross-reactivity [5].

Most of the studies about Brazilian sand flies and their vertebrate food sources demonstrated a predominant orientation towards the use of DNA-based methods, although Sanger sequencing, Polymerase chain reaction–restriction fragment length polymorphism (PCR-RFLP), or specific primers for each host. Studies have employed exclusively mitochondrial targets, such as Cytochrome Oxidase I (COI) [6] and the nuclear gene prepronociceptin (PNOC) [7]. Notably, Cytochrome B (CytB) was the preferred molecular marker [5].

The molecular identification of phlebotomine sand flies’ bloodmeals has become a valuable tool for elucidating the transmission dynamics of leishmaniasis [4]. Nevertheless, there is a barrier to detecting mixed feeding when Sanger sequencing was employed due to the challenge of identifying polymorphic sites, since multiple dietary sources within the amplicon generate overlapping chromatogram peaks, and the Sanger method cannot reliably resolve such base-calling ambiguities [5].

Advancements in sequencing technologies have enabled the DNA metabarcoding approach, allowing the detection of multiple species present within a single sample [8]. The DNA obtained from the stomach or gut content of invertebrates that feed on vertebrates, defined as invertebrate-derived DNA or ingested-derived DNA (iDNA), can be used to detect vertebrate species as food sources as a promising tool for interaction studies [9,10,11,12,13,14].

To date, few studies have applied NGS technologies to the identification of bloodmeal sources taken by field-collected phlebotomine sand flies. DNA metabarcoding has enabled the identification of multiple species present in the stomach and gut content of invertebrates by mass-PCR amplification and high-throughput sequencing [9,12,13]. Therefore, DNA metabarcoding sequencing is suggested to be preferred in studies investigating host–vector interactions in the Neotropical region [4].

Despite their significance as vectors of *Leishmania* spp., studies on the feeding behaviors of sand flies can contribute to the development of more effective vector control strategies, ultimately helping to mitigate the burden of leishmaniasis in endemic regions, particularly in Brazil [5].

The Cacao Region, in the state of Bahia, Brazil, is partially covered by the Brazilian Atlantic rainforest, and presents a remarkable wild mammal diversity that is involved in a complex transmission cycle of *Leishmania* (*Viannia*) *braziliensis* Vianna, 1911. This major spatial circuit of American Tegumentary Leishmaniasis (ATL) endemicity in Northeast Brazil presents a ‘Modified Silvatic’ eco-epidemiological profile [15]. The enzootic cycle of *L*. (*V*.) *braziliensis* within this circuit occurs between wild mammals in a forested environment, where transmission of the parasite among hosts is carried out by exophilic sand fly species. This enzootic cycle spills over to the zoonotic peridomiciliary transmission of *L*. (*V*.) *braziliensis* among human beings and domestic and synanthropic mammals, involving mainly the endophilic phlebotomine sand fly species [16].

It was previously reported that human cases of ATL in the Cacao Region occur as time–space clusters, shaping an endemic maintained by concurrent and successive outbreaks of ATL [17]. Especially among acute forms of ATL, such as Cutaneous (CL) and Disseminated Leishmaniasis (DL), close temporal-geographic proximity of individuals to a recently diagnosed case of CL or DL caused them a significant risk of developing the disease [17,18]. This indicates the local aggregation of the transmission determinants but also suggests that humans may be relevant actors maintaining the observed outbreaks.

In the present study, we identified the vertebrate species potentially involved in the transmission cycle of ATL by assessing iDNA of female sand flies with the DNA metabarcoding approach in the Cacao Region, where an ongoing outbreak was detected. We performed a high-throughput sequencing of two mini-barcodes [19,20] and a traditional barcode [21] to compare their efficiency. We also related phlebotomine sand flies naturally infected with *L*. (*V*.) *braziliensis* with vertebrate species found in its putative food sources.

## 2. Materials and Methods

### 2.1. Study Area

This study was carried out in a hyper-endemic region for American Tegumentary Leishmaniasis (ATL) caused by *Leishmania* (*Viannia*) *braziliensis*, located in the Cacao Region of the Bahia state, Brazil [22]. The Cacao Region comprises 20 municipalities within a rural area in the southeast of Bahia, northeast Brazil. Residents of this area are exposed to the ATL during their labor activities, mostly in agriculture, such as cacao, banana, and clove crop plantations, which are often carried out in primary or secondary forests spread within the Atlantic rainforest biome [16,23].

### 2.2. Sample Collection and Processing

Phlebotomine sand flies were captured near nine residences of ATL cases between May 2018 and June 2019 (ICMBio-MMA n°71661-1) via Entomological surveys performed by the Brazilian Ministry of Health [24].

Zoological material was collected using night-time light traps of the Centers for Disease Control and Prevention (CDC) in the forest and anthropic environments (peridomestic environment and inside human dwellings). We installed three traps per residence (CAAE: 07676319.0.0000.0049) at a height of 1 m for periods of 12 h, from 6:00 p.m. to 6:00 a.m.: two in the home environment (dormitories, external walls of the house, and shelters of animals) and one in the wild environment (extradomiciliary, forest edge up to 500 m from the residence). All the material collected was placed in polypropylene tubes and cryopreserved (−20 °C) in 70% alcohol.

The thorax and the first abdominal segment of each female sand fly were dissected and cryopreserved (−20 °C) in Dimethyl sulfoxide (DMSO) at 6% for molecular analysis; the head and the last abdominal segment were mounted between slides/coverslips, which were fixed in Hoyers’ solution for species identification. Taxonomic analysis was performed adopting Young and Duncan [25], and Galati [26], and the abbreviations of the sand fly genera are those proposed by Marcondes [27].

Samples were separated according to collection date, ecotype (intradomiciliary, peridomiciliary, and extradomiciliary), and taxonomic identification; a measure that aimed to track the information of each sample. Thus, female sand flies collected were organized individually or into pools of up to fourteen insects; some were engorged, indicating a recent feeding, and were separated to identify their bloodmeals by the iDNA metabarcoding analyses. We also included the non-engorged female sand flies naturally infected with *L*. (*V*.) *braziliensis* from previous studies by our research group [16] to evaluate the sensitivity of the metabarcoding approach for identifying bloodmeals.

### 2.3. iDNA Metabarcoding for Bloodmeal Identification

The iDNA present in the thorax and the first abdominal segment dissected from the sand flies was extracted using the Invitrogen Genomic DNA Mini Kit^®^ (Waltham, MA, USA), following the manufacturer’s protocol.

For the identification of the species present in the bloodmeal, we used a 305 bp barcode region of the CytB gene [21]. In addition to this, two mini-barcodes were also used: the 16S rRNA (134 bp) [19], a mammal-specific marker, and the 12S rRNA (139 bp) [20], a broader vertebrate-specific marker. As suggested by the literature [28], we changed the first nucleotide to a degenerate base (5′-Y) for the 12S primer to allow broader binding in more mammal species.

For the sequencing assay used to characterize the mammal diversity in phlebotomine sand fly samples, a first PCR using the primers for the CytB was conducted following the PCR protocol from Kocher [21]. CytB amplicon samples were forwarded individually to MiSeq Illumina^®^. For 12S rRNA and 16S rRNA amplification, we followed the protocols of Saranholi [12]. We utilized human-specific blocking primers for 12S rRNA [14] and 16S rRNA [29] to improve the sensitivity of high-throughput sequencing and reduce the amplification of human DNA. Moreover, we incorporated a 5-nucleotide barcode unique identifier (tags) in both forward (F) and reverse (R) primers to mark each sample of PCR products and reduce the sequencing cost [11,12].

For metabarcoding sequencing of the three molecular markers, the PCR products were purified using magnetic beads (1.2 μL Agencourt AMPure XP^®^ Beckman Coulter per 1 μL of PCR product), quantified with a Qubit fluorimeter (Thermo Fisher, Waltham, MA, USA), and normalized to 50 ng/μL. Indexing was performed using the Nextera Index kit^®^ (Illumina, San Diego, CA, USA). CytB amplicon samples were forwarded individually to PCR amplification for incorporating the Illumina adaptors, and the barcoded CytB amplification products from female sand flies were paired-end sequenced on an Illumina MiSeq instrument^®^. For mini-barcodes 12S rRNA and 16S rRNA, paired-end sequencing was carried out on the Illumina iSeq^®^ platform, using the iSeq 100 v2 300 cycles reagent kit (2 × 150 bp, Illumina, San Diego, CA, USA), generating a total of 70,000–100,000 reads per metabarcoding sample.

### 2.4. Metabarcoding Bioinformatics Analysis

The raw data obtained were first analyzed to check the sequencing quality via FastQC^®^ v. 0.12.1 software [11]. The resulting sequences of MiSeq^®^ (CytB) and iSeq^®^ (12S and 16S) paired-end runs were demultiplexed using the process_radtags program in Stacks^®^, to trace back the information of each sand fly species and the sampling point locality [12]. Following Rodgers et al. [30], we merged the corresponding forward and reverse sequences and trimmed them to a minimum quality score threshold (−q) of 15, a minimum overlap (−v) of 150 bp, and a minimum length (−n) of 200 bp, using PEAR^®^ v.0.9.6 [31]. We discarded all singletons and obtained the OTUs (Operational Taxonomic Units) by clustering the reads with at least 97% similarity using USEARCH v.11.0.667^®^ [32].

The obtained OTUs were compared with reference sequences available in the GenBank NCBI^®^, via Nucleotide BLAST (Basic Local Alignment Search Tool; https://blast.ncbi.nlm.nih.gov/Blast.cgi, accessed on 20 March 2024) to identify sand flies’ bloodmeal species. The criteria of a high percentage of matches (98–100%) were used for OTU assignment at the species level, combined with referencing geographic databases [11]. When a sequence had a match for two or more species, we defined the species according to its expected occurrence in the study area using IUCN^®^—International Union for Conservation of Nature and GBIF^®^ Occurrence. Available online: https://www.gbif.org/occurrence/search (accessed on 20 March 2024).

Faced with a high percentage of matches obtained for the species that do not occur in the area, we assigned the OTU to the species from the same genus with natural occurrence in the region. For matches between 90 and 97.99%, we assumed the genus, family, or order assignment, and matches with less than 90% of similarity were removed. Finally, OTUs with relative abundance lower than 0.05% (<8 reads) within each tagged amplification were also removed [11,12].

## 3. Results

We analyzed forty female sand flies from seven species, distributed in thirteen samples. Proven or putative ATL vectors in Brazil were analyzed: *Ny. whitmani*, *Mg. migonei*, *Pi. fischeri*, and *Ps. hirsutus*; besides exophilic species: *Ev. bahiensis*, *Ty. longispina*, and *Th. viannamartinsi*. We included two intradomiciliary samples, four from peridomiciliary, and seven from extradomiciliary. Four samples are composed of four to thirty-one female sand flies naturally infected with *L*. (*V*.) *braziliensis* from previous studies by our research group [10], three of them with pools of 03, 13, and 14 female sand flies (Table 1).

Mini barcodes 12S rRNA and 16S rRNA were successfully amplified in 10 samples with seven thousand reads per sample. CytB-barcode was amplified in eight samples with eighty to three hundred thousand reads per sample. We are able to amplify these three regions for five samples (Table 1).

Metabarcoding results revealed a total of 22 OTUs, and we identified 11 different OTUs for each molecular marker adopted, with 30 and 61 detections for mini-barcodes 12S rRNA and 16S rRNA, respectively, and 29 detections for CytB-barcode. From the total of the OTUs detected, 20 OTUs were from vertebrate species, including primates, rodents, ungulates, marsupials, reptiles, and amphibians as possible sand flies’ bloodmeals: eight vertebrate orders for *Th. viannamartinsi* (14 OTUs); followed by *Mg. migonei* with seven vertebrate orders detected in 14 OTUs; five vertebrate orders for *Ny. whitmani* (09 OTUs); four vertebrate orders for *Pi. fischeri* (04 OTUs) and *Ty. longispina* (09 OTUs); three vertebrate orders for *Ps. hirsutus* (08 OTUs) and *Ev. bahiensis* (03 OTUs) (Table 2 and Table 3).

Mammal species detected include primates such as *Alouatta guariba*, *Brachyteles arachnoides,* and *Sapajus xanthosternos*; ungulates as domestic animals, horse, donkey, pig, and ox, besides other ungulates such as deer—*Mazama* sp.; rodent mammals as paca—*Cuniculus paca*, Brazilian porcupine—*Chaetomys subspinosus* or *Coendou prehensilis*, and bush mouse—*Trinomys albispinus*. Domestic dog, and marsupial species—*Gracilinanus microtarsus* were also detected, other than chickens (Table 3).

In the 12S rRNA, we also observed iDNA from three amphibian species—*Chiasmocleis schubarti*, *Dendropsophus elegans,* and *Phyllomedusa bahiana*. CytB results show us iDNA from reptile species, such as sucuri snakes—Eunectes murinus and OTUs of Phlebotomies’ genus, *Nyssomyia* and *Trichophoromyia* (Table 2 and Table 3).

*Bos taurus*, *Sapajus xanthosternus*, and *Sus scrofa* were identified in samples with female sand flies naturally infected with *L*. (*V*.) *braziliensis*, of the species *Ny. whitmani*, *Ps. hirsutus*, and *Ty. longispina*; *Equus asinus*, in samples of *Th. viannamartinsi*; *Gracilinanus microtarsus* in *Ty. longispina*; *Equus caballus* in *Ny. whitmani*; and *Alouatta guariba* in *Ps. hirsutus* (Table 2). All these samples of phlebotomine sand flies naturally infected with *L*. (*V*.) *braziliensis* were obtained during a single capture in two residences of ATL cases in the municipality of Taperoá, Cacao Region, Brazil [16].

*Homo sapiens* was detected in all samples by all discrimination techniques employed in the study. This was not preventable even using human-specific blocking primers [14,29]. *Canis lupus*, *Equus asinus*, and *Equus caballus* were the sand flies’ bloodmeals identified by the three molecular markers used, presenting the highest ratio of detection (Table S1). CytB was more effective for detecting rodents (five of the eight detections) and *C. lupus* (four of the seven detections); 16S rRNA for wild primates and ungulates; 12S rRNA for *G. gallus* (six of the eleven detections), marsupials, and amphibians (Table 3).

Among domestic animals, *C. lupus* and *E. asinus* were identified in *Mg. migonei*, and *Th. viannamartinsi* in this study. We detected *Equus caballus* in *Mg. migonei*, *Ny. whitmani*, and *Th. viannamartinsi*. *Gallus* was identified in *Ev. bahiensis*, *Mg. migonei*, *Ny. whitmani*, *Pi. fischeri*, *Ty. longispina,* and *Th. viannamartinsi*. We identified *Sus scrofa* in *Mg. migonei*, *Ny. whitmani*, *Pi. fischeri*, *Ps. hirsutus*, *Ty. longispina,* and *Th. viannamartinsi* (Table 2).

*Alouatta guariba* was detected in *Ps. hirsutus*, and *Sapajus xanthosternus* in *Ny. whitmani*, *Ps. hirsutus*, *Th. viannamartinsi*, and *Ty. longispina*. From detected Rodentia species recognized as putative *Leishmania* spp. hosts, *Coendou prehensilis* and *Cuniculus paca* in *Mg. migonei;* furthermore, *Trinomys albispinus* was detected in *Ev. bahiensis*. Among Didelphimorphia, we detected *Gracilinanus microtarsus* in *Th. viannamartinsi* and *Ty. longispina*. We detected frogs in *Mg. migonei*, *Ny. whitmani*, *Ps. hirsutus*, *Th. viannamartinsi*, and *Ty. longispina*, and sucuri snakes in *Th. viannamartinsi* (Table 2).

## 4. Discussion

DNA metabarcoding approach identified sand flies’ bloodmeal with a high sensitivity, since this technique promoted the detection of twenty vertebrate species. Among these species, nine are recognized as proven or putative hosts of *Leishmania* spp.: *Alouatta guariba*, *Bos taurus*, *Canis lupus*, *Coendou prehensilis*, *Cuniculus paca*, *Equus asinus*, *Equus caballus*, *Gracilinanus microtarsus*, *Sapajus xanthosternus*, and *Sus scrofa* [5,33,34,35,36].

In previous studies, *Alouatta guariba* and *Sapajus xanthosternus* were found positive for *Leishmania* (*Leishmania*) *infantum* Nicolle, 1908, despite not showing clinical signs of Visceral Leishmaniasis [37]. *Alouatta seniculus* was also detected in *Ps. hirsutus*, and *Nyssomyia* species from the Western Brazilian Amazon; however, this primate is not yet considered a host/reservoir of *Leishmania* spp. [38].

Rodentia is a heterogeneous mammalian taxon that presents a spectrum of competence as reservoirs of the genus *Leishmania*, including tolerant species with high infectivity for the vector and species that rapidly eliminate the infection [34]. *Leishmania* (*Leishmania*) sp. infection was confirmed in *Coendou* sp. in the Guiana Amazonian [39]; *Cuniculus paca* was found naturally infected with *Leishmania* (*Viannia*) *lainsoni* (Silveira et al., 1987) in the Amazonian Brazilian state of Pará [40]. Representatives of *Proechimys* seem to be hosts of dermotropic species of *Leishmania* in sylvatic areas from Amazonian [41]. In addition, isolates of *Leishmania* sp. were recovered from specimens of *Trinomys iheringi* (Thomas, 1911) (sin. *Proechimys iheringi denidsgratus* Moojen) captured in the endemic ATL region of Atlantic Forest in Bahia, Brazil [42], near our study area, where we found *Trinomys albispinus* as sand fly bloodmeal in *Ev. bahiensis*.

In general, opossums are frequently found in peridomiciliary, where they may serve as a source of infection to leishmaniasis vectors; thus, Didelphimorphia have also been suspected of sustaining *Leishmania* spp. infections [5]. *Gracilinanus microtarsus* predominates in forested environments, occasionally occurring in open agricultural areas due to habitat fragmentation. The natural infection of this marsupial by *Leishmania* spp., including *L. (L.) infantum*, in south Bahia is a recent record in Brazil [43].

We detected five domestic animal species in iDNA from sand fly bloodmeals: *Bos taurus*, *Canis lupus*, *Equus asinus*, *Equus caballus*, and *Sus scrofa*. The dog plays a prominent role due to its proximity to humans, acting as a link in the *Leishmania* spp. transmission cycle, since this domestic animal was detected as a bloodmeal in thirteen sand fly species in Brazil [5]. This animal has been found naturally infected by dermotropic species in endemic areas, incriminated as a possible domestic reservoir of *L*. (*V*.) *braziliensis* in the Cacao Region, Bahia, Brazil [44].

Among the horses, donkeys, and hybrids involved with ATL, several studies have suggested the natural infection by *L*. (*V*.) *braziliensis* [45,46,47]. In the Cacao Region, Bahia, a donkey, *Equus asinus*, was found naturally infected with *L*. (*V*.) *braziliensis* through parasites isolated from a lesion located on a castration scar and identified using monoclonal antibodies [48]. Artiodactyls and Equines may not be considered primary hosts of *Leishmania* parasites, but their presence can influence sand fly density and human-sand fly exposure, thereby affecting leishmaniasis transmission dynamics [49].

Domestic pigs and chickens were detected in six of the seven phlebotomine sand fly species analyzed in this study. *Sus scrofa* has been found infected by *Leishmania* sp., but only in serological reactions in the endemic ATL area in Bahia, Brazil [50].

*Gallus gallus* is not a reservoir of the *Leishmania* spp. because it is refractory to parasite infection, despite being a food source for species of phlebotomine sand fly ATL vectors [51]. Phlebotomine sand flies that feed on various bird orders may indicate their ecological habits, such as utilizing tree canopies for opportunistic blood feeding [5]. In our study area, chickens take a rest on a cacao tree in the peridomiciliary, which can increase the risk of ATL contagion for humans and other animals, since this domestic animal was detected in 25 sand fly species in Brazil [5]. The presence of a chicken coop may attract sand flies, thereby increasing the chances of humans being bitten by them, but, contradictorily, if the vector feeds on chickens, it could reduce the proportion of effective bites on persons [51].

Human blood is the food source identified in at least 35 sand fly species from Brazil [5]. Our finding on the predominance of human DNA across all iDNA samples analyzed reinforces the observations mentioned above for other Brazilian regions. Just as noteworthy, while the current study falls short of providing evidence proving the existence of a strong anthroponotic component in the transmission cycle of *L*. (*V*.) *braziliensis*, it does shed light on the prominent role played by humans in the outbreaks that evolve into the endemics of ATL. On the other hand, the possibility of cross-contamination of samples with human DNA during sand fly processing should be taken into consideration, although we used an iDNA sterile room for PCR, reducing the risk of bias in sample quality [5].

Some mammal species detected as food sources of phlebotomine sand flies collected in this work must be observed with caution for ATL transmission in the Cacao Region, Bahia, Brazil. There is no consensus on whether *Brachyteles arachnoides*, *Chaetomys subspinosus*, *Mazama* sp., and *Trinomys albispinus* act as accidental hosts, which play no proven role in the ATL transmission cycle [34,35], in this endemic area.

The encounter of an infected animal is not enough to consider it a reservoir. The competence of the animal species in maintaining and/or transmitting the parasite will define its role as a reservoir. An infected animal is a host of the parasite, but its importance in maintaining the transmission cycle in a given area will depend on the peculiarities of the parasite–host interaction. In a reservoir of leishmaniasis, an infectious agent survives persistently in a way that the animal may serve as a source of parasites to the vectors in the same habitat [34,35].

Phlebotomine sand flies may be affected by changes in local mammal communities, partially driven by the abundance and composition of vertebrate fauna [52]. Our results demonstrated opportunistic species circulating in patients’ ATL homes and feeding on susceptible mammals, thus maintaining a zoonotic peridomiciliary transmission cycle of this etiologic agent.

Non-mammal species, such as sucuri snake (*Eunectes murinus*) and amphibians (*Chiasmocleis schubarti*, *Dendropsophus elegans*, and *Phyllomedusa bahiana*), may help maintain the enzootic cycle of *Leishmania* spp. in forested environments because these animals can act as accessory food sources for sand fly species, which are also capable of feeding on humans or animals infected by *L*. (*V*.) *braziliensis*. In addition, our detection of non-mammalian blood sources highlights important ecological considerations for ATL transmission. Although these taxa are not established as competent reservoirs for *Leishmania* spp., they may influence transmission dynamics by sustaining local sand fly populations, altering host availability. Their presence in bloodmeals not only suggests feeding plasticity and use of different forest microhabitats, but may also produce novel contact patterns among vectors, humans, domestic animals, and wildlife. For example, studies employing iDNA metabarcoding in Amazonia documented feeding on diverse vertebrate taxa (including non-mammals) in sand flies, linking host–vector interactions to deforestation gradients [13]. Nevertheless, certain vertebrate groups may act as attractants for sand flies without supporting parasite development, thus increasing human exposure risk, especially in the case of domestic species (e.g., domestic chicken [51]).

Although the detection of non-mammalian blood sources likely reflects genuine feeding plasticity of several sand fly species in forested and peridomestic environments, it is also important to consider methodological factors that may influence these results. In metabarcoding studies, low concentrations of DNA from environmental exposure or contact with other organisms may occasionally be co-amplified, particularly when dealing with very small-bodied insects such as phlebotomine sand flies, whose limited bloodmeal volume increases in relative impact of trace DNA. While our workflow included strict contamination-control procedures (dedicated iDNA sterile room, physical separation of pre- and post-PCR steps, use of disposable tools), the possibility of rare background amplification cannot be fully excluded. To minimize the impact of cross-contamination, in addition to taking precautions during laboratory procedures, it is important to include control samples during amplification steps and be careful in filtering reads on bioinformatic analyses [53].

Nonetheless, there are records of frogs as blood feed in phlebotomine sand flies, *Sergentomyia* França and Parrot, 1920, in South Africa [54] and India [55]. This genus preferably feeds on small reptiles and amphibians [55], and it was involved with the transmission cycle of *Leishmania* (*Sauroleishmania*) spp. (Lainson and Shaw, 1987), associated with snakes in China [56]. To our knowledge, *Mg. migonei*, *Ny. whitmani*, *Ps. hirsutus*, *Th. viannamartinsi*, and *Ty. longispina* feeding on amphibians and *Th. viannamartinsi* feeding on snakes have not yet been reported in the literature. Anurans of the family Hylidae—*Bokermannohyla martinsi* Bokermann, 1964, and *Scinax fuscovarius* (Lutz, 1925) were identified as a blood source by gene sequencing of COI in *Sciopemyia* spp. collected inside ferruginous caves located at “Quadrilátero Ferrífero”, Minas Gerais [7].

Several unexpected species were found as sand fly bloodmeal through the iDNA metabarcoding approach. The less competent vertebrate species can help decrease the density of reservoirs; thus, *Leishmania* parasites may indeed end up inside “diluting” hosts. This mechanism of potential regulation of pathogens in natural ecosystems, driven by host species, is inefficient for *Leishmania* spp. transmission is referred to as the “dilution effect” [57]. Therefore, the biodiversity loss impacts leishmaniasis transmission, since a positive correlation exists between the relative abundance of reservoir hosts and *Leishmania* spp. prevalence rate in sand flies [52].

A “dilution effect” in ATL can be expected if sand fly vectors feed on various host species, including some poorly competent for *L*. (*V*.) *braziliensis* transmission. We observed in our area of the Brazilian Atlantic Forest a similar scenario to that of Amazonian leishmaniasis in French Guiana, since sand fly vectors fed on different hosts, including some not known as *Leishmania* reservoirs [52].

*Leishmania* parasites circulate among wild mammals in Atlantic Forest fragments in southern Bahia, posing transmission risks to humans and domestic animals near these remnants. As deforestation continues, increased wildlife–domestic–human interactions will heighten zoonotic transmission risk [43]. There must be a higher vector density for the transmission of *L*. (*V*.) *braziliensis* to humans and other domestic animals [34,35].

Our results contribute to studies about the ecology of ATL sand fly vectors in the Cacao Region, Bahia, Brazil. The feeding preferences of *Th. viannamartinsi* and *Mg. migonei* are undoubtedly notable, since we detected fourteen bloodmeal sources, associated with eight and seven vertebrate orders, respectively, in only three samples with individual female sand flies for each of these species. Comparing these results with those found for *Ny. whitmani* in this study, three samples with 28 females were analyzed, and we detected nine bloodmeal sources associated with five vertebrate orders.

Dutra–Rêgo et al. [5] identified eight vertebrate orders associated with food sources in *Ny. whitmani*, demonstrating a high plasticity of these species in feeding habits; *Ps. hirsutus* and *Mg. migonei* are associated with four vertebrate orders; and *Pi. fischeri* is associated with only one vertebrate order. This systematic review revealed one, two, and seven vertebrate orders as bloodmeals in sand fly genera *Trichopygomyia*, *Trichophoromyia*, and *Evandromyia*, respectively. This study reported at least ten vertebrate species as bloodmeals in *Psychodopygus* spp. and suggested studies to ascertain *Evandromyia* spp. involvement in the leishmaniasis epidemiological cycle [5].

The feeding plasticity in *Th. viannamartinsi* and *Mg. migonei*, including humans as a bloodmeal, is perhaps linked to a high dispersal or quantity of gonotrophic cycles of its female sand flies, suggesting that maybe the first species has vectorial capacity [16], since the second acts as an accessory ATL vector [58,59]. Anthropophilic behavior does not necessarily confirm a species vector; thus, studies are necessary to evaluate the capacity and competence vectorial of suspected species such as *Th. viannamartinsi*, particularly in the Cacao Region, Bahia, Brazil. At least six sand fly species analyzed in this study are recognized as proven ATL vectors in the Cacao Region, Bahia [58], which can result in higher *L*. (*V*.) *braziliensis* transmission due to functional complementarity between vector species, *Ny. whitmani*, *Mg. migonei*, *Pi. fischeri*, and *Ps. hirsutus* [60].

The iDNA metabarcoding approach was successful in detecting mixed feeding in sand fly ATL vectors collected from intradomiciliary, peridomiciliary, and extradomiciliary residences of ATL cases, and some of these female sand flies were infected by *L*. (*V*.) *braziliensis*. Dutra–Rêgo et al. [5] reported four species of the *Nyssomyia* genus with mixed feeding through ELISA/precipitin techniques, and PCR-RFLP; three of *Evandromyia* spp., and two of *Psychodopygus* spp.

Massey et al. [13] demonstrated the potential of metabarcoding to disentangle complex trophic networks by detecting a wide diversity of host species from insect bloodmeals, highlighting its value for understanding transmission dynamics in vector-borne disease systems. The disadvantages are the high equipment and sample-processing costs, the necessity of specialized training for data analysis, and the fact that partially digested DNA cannot be sequenced. Targeted enrichment before NGS can also be beneficial for the enhancement of microscopic quantities of DNA present in bloodmeals [4]. It can be carried out by PCR amplification of the desired loci, as we did in this work.

Sand flies present substantial methodological constraints in molecular research due to their small corporeal dimensions, which compromise the ability to obtain optimal DNA concentrations needed for effective amplification of designated molecular targets. When considering sand flies that have fed on various vertebrates, the amount of blood ingested from each source can vary, influencing DNA recovery and consequently limiting the ability to identify mixed feedings [61]. Standardized experimental protocols and careful monitoring of storage parameters and blood digestion status in female sand flies are essential for improving our knowledge of sand fly feeding ecology in ATL transmission dynamics.

Numerous studies of sand fly feeding tendencies are limited, as they do not always identify the actual bloodmeal sources. A limitation of barcoding analyses was the absence of reference sequences for some sand fly bloodmeal sources from the Cacao region in GenBank^®^ NCBI. In cases where exact species matches were unavailable for mini-barcodes (12S rRNA and 16S rRNA) or CytB, the sequences matched with congeneric species, as observed with *Mazama* sp., possibly *M*. *gouazoubira*. Additionally, CytB lacked specificity for vertebrates, leading to competition for reads during metabarcoding and matches with *Nyssomyia* sp. and *Trichophoromyia* sp. in OTU clustering.

Despite these challenges, our methodology was sensitive enough to identify mixed bloodmeals from a single non-engorged female sand fly infected with *L*. (*V*.) *braziliensis*. The three markers used in this study showed complementary strengths in recovering vertebrate diversity from iDNA samples. The 16S rRNA gene was the most sensitive, yielding the highest number of detections and proving particularly effective for terrestrial mammals, such as *Brachyteles arachnoides* and *Chaetomys subspinosus*. The 12S rRNA gene was critical for broadening taxonomic coverage by identifying not only eutherian mammals but also marsupials (*Gracilinanus microtarsus*), other than birds (*Gallus gallus*) and amphibians (*Chiasmocleis schubarti*, *Dendropsophus elegans*, and *Phylomedusa bahiana*). CytB contributed unique and robust identifications for sucuri snakes—*Eunectes murinus*; rodents (*Chaetomys subspinosus* and *Trinomys albispinus*); and domestic dog (*Canis lupus familiaris*), making it valuable for assessing epidemiological interfaces between wildlife, domestic species, and humans. Taken together, the combined use of 12S, 16S, and CytB markers enhanced taxonomic resolution and reliability, underscoring the importance of a multi-marker approach for elucidating host–vector interactions in the Neotropical region.

Previous studies in Neotropical systems have already highlighted that applying multiple markers improves the recovery of vertebrate diversity from iDNA samples [11,12], and our findings further reinforce this pattern. Therefore, diversifying molecular targets in iDNA metabarcoding could improve the identification of sand fly bloodmeal sources in endemic ATL regions, such as the Cacao Region in Bahia, Brazil.

## 5. Conclusions

DNA Metabarcoding to determine the species represented in mixed biological samples offers a compelling opportunity to mine vast amounts of sequence data from individual samples, including identifying pathogens, hosts, and vectors from a single analysis. These results demonstrated opportunistic species with circulation at patients’ ATL homes newly diagnosed feeding on *L*. (*V*.) *braziliensis* susceptible mammals, thus maintaining a zoonotic peridomicililary transmission cycle of this etiologic agent. This molecular approach allowed us to accurately sample the diversity of phlebotomine sandflies’ bloodmeals in a single specimen of a non-engorged female sand fly with mixed feeding. In addition, by combining molecular markers for iDNA metabarcoding, this approach can enhance vector surveillance programs, support risk assessment for pathogen transmission, and inform more effective strategies for controlling zoonotic diseases in endemic regions.

## Figures and Tables

**Table 1:** Phlebotomine sand fly species collected between May 2018 and June 2019 in the Cacao Region, Bahia, Brazil. Sample data were stratified by reads per sample according to barcode or mini-barcode, species, collection locality, and capture ecotype: intradomiciliary, peridomiciliary, and extradomiciliary.

Sample	Species	♀	Reads per Sample According to Barcode or Minibarcodes			Municipality of Bahia-Brazil	Ecotope
			12S rRNA	16S rRNA	CytB		
M3C1PA	*Nyssomyia whitmani*	13 *	7000	7000		Taperoá	Peridomiciliary
M3C1EC	*Trichopygomyia longispina*	01 *	7000	7000		Taperoá	Extradomiciliary
M3C2EA	*Nyssomyia whitmani*	14 *	7000	7000		Taperoá	Extradomiciliary
M3C2EB	*Psychodopygus hirsutus*	03 *	7000	7000		Taperoá	Extradomiciliary
M2C1I2	*Nyssomyia whitmani*	01	7000	7000	90,000	Teolândia	Intradomiciliary
M3C2I	*Trichophoromyia viannamartinsi*	01	7000	7000	300,000	Taperoá	Intradomiciliary
M4C2E	*Trichophoromyia viannamartinsi*	01	7000	7000	274,000	Wenceslau Guimarães	Extradomiciliary
M5C1E	*Migonemyia migonei*	01	7000	7000	80,000	Gandu	Extradomiciliary
M6C2E	*Migonemyia migonei*	01	7000	7000	80,000	Gandu	Extradomiciliary
M7C2P1	*Migonemyia migonei*	01	7000	7000		Presidente Tancredo Neves	Peridomiciliary
M3C1PB	*Trichophoromyia viannamartinsi*	01 *			80,000	Taperoá	Peridomiciliary
M6C3P	*Pintomyia fischeri*	01			90,000	Gandu	Peridomiciliary
M9C2E	*Evandromyia bahiensis*	01			90,000	Presidente Tancredo Neves	Extradomiciliary

* Sample with at least one female non-engorged phlebotomine sand fly infected by *Leishmania* (*Viannia*) *braziliensis*.

**Table 2:** iDNA metabarcoding results from samples of phlebotomine sand flies collected between May 2018 and June 2019 in the Cacao Region, Bahia, Brazil. Operational taxonomic units (OTUs) according to the barcode (CytB) or the mini barcodes (12S rRNA and 16S rRNA).

Sand Fly Species	Samples	OTUs per Sample According to Mini-Barcodes or Barcode		
		12S rRNA	16S rRNA	CytB
*Trichophoromyia viannamartinsi* (Sherlock e Guitton, 1970)	03	*Homo sapiens* *Canis lupus* *Gallus gallus* *Equus asinus* *Equus caballus* *Gracilinanus microtarsus* *Phylomedusa bahiana*	*Homo sapiens* *Bos taurus* *Canis lupus* *Equus asinus* *Equus caballus* *Sus scrofa* *Mazama* sp. *Brachyteles arachnoides* *Sapajus xanthosternus*	*Homo sapiens* *Canis lupus* *Gallus gallus* *Equus asinus* *Eunectes murinus* *Chaetomys subspinosus* *Nyssomyia* sp. *Trichophomyia* sp.
*Migonemyia migonei* (França, 1920)	03	*Homo sapiens* *Gallus gallus* *Equus asinus* *Coendou prehensilis* *Cuniculus paca* *Chiasmocleis schubarti*	*Homo sapiens* *Bos taurus* *Canis lupus* *Equus asinus* *Equus caballus* *Sus scrofa* *Cuniculus paca* *Mazama* sp. *Brachyteles arachnoides Sapajus xanthosternus*	*Homo sapiens* *Canis lupus* *Gallus gallus* *Equus asinus* *Equus caballus* *Sus scrofa* *Chaetomys subspinosus* *Nyssomyia* sp.
*Nyssomyia whitmani* (Antunes e Coutinho, 1939)	03	*Homo sapiens* *Gallus gallus* *Dendropsophus elegans*	*Homo sapiens* *Bos taurus* *Equus caballus* *Sus scrofa* *Mazama* sp. *Sapajus xanthosternus*	*Homo sapiens* *Sus scrofa* *Nyssomyia* sp.
*Psychodopygus hirsutus* (Mangabeira, 1942)	01	*Chiasmocleis schubart Dendropsophus elegans*	*Homo sapiens* *Bos taurus* *Sus scrofa* *Mazama* sp. *Alouatta guariba* *Sapajus xanthosternus*	
*Pintomyia fischeri* (Pinto, 1926)	01			*Homo sapiens* *Gallus gallus* *Sus scrofa* *Chaetomys subspinosus* *Nyssomyia* sp.
*Evandromyia bahiensis* (Mangabeira e Sherlock, 1971)	01			*Homo sapiens* *Gallus gallus* *Trinomys albispinus* *Nyssomyia* sp.
*Trichopygomyia longispina* (Mangabeira, 1942)	01	*Homo sapiens* *Gallus gallus* *Dendropsophus elegans* *Gracilinanus microtarsus*	*Homo sapiens* *Bos taurus* *Sus scrofa* *Mazama* sp. *Brachyteles arachnoides* *Sapajus xanthosternus*	
Total	13	11 OTUs	11 OTUs	11 OTUs

**Table 3:** Taxonomic identification of phlebotomine sand fly bloodmeals detected via iDNA metabarcoding from samples collected between May 2018 and June 2019 in the Cacao Region, Bahia, Brazil. Vertebrate species were presented according to class and order.

Taxon ID	Number of Taxon Detections According to Mini-Barcodes or Barcodes from the Total of the Samples Analyzed		
Mammalia	12S rRNA	16S rRNA	CytB
Primates			
*Alouatta guariba* Humboldt, 1812		01	
*Brachyteles arachnoides* Geoffroy, 1806		04	
*Homo sapiens* Linnaeus, 1758	08	10	08
*Sapajus xanthosternus* Wied–Neuwied, 1826		10	
Artiodactyla			
*Bos taurus* Linnaeus, 1758		10	
*Mazama* sp. Rafinesque, 1817		10	
*Sus scrofa* Linnaeus, 1758		08	03
Rodentia			
*Coendou prehensilis* Linnaeus, 1758	01		
*Chaetomys subspinosus* Olfers, 1818			04
*Cuniculus paca* Linnaeus, 1766	01	01	
*Trinomys albispinus* Geoffroy, 1838			01
Carnivora			
*Canis lupus familiaris* Linnaeus, 1758	01	02	04
Didelphimorphia			
*Gracilinanus microtarsus* Wagner, 1842	02		
Perissodactyla			
*Equus asinus* Linnaeus, 1758	02	02	02
*Equus caballus* Linnaeus, 1758	01	03	01
Birds			
*Gallus gallus* Linnaeus, 1758	06		05
Reptilia			
*Eunectes murinus* Linnaeus, 1758			01
Anura			
*Chiasmocleis schubarti* Bokermann, 1952	02		
*Dendropsophus elegans* Wied–Neuwied, 1824	05		
*Phylomedusa bahiana* Lutz, 1925	01		
Total	30	61	29

## Data Availability

The original contributions presented in this study are included in the article/Supplementary Materials. Further inquiries can be directed to the corresponding author.

## Supplementary Materials

The following supporting information can be downloaded at: https://www.mdpi.com/article/10.3390/microorganisms13122650/s1. Table S1: Vertebrate species identified from sand fly females collected between May 2018 and June 2019 in the Cacao Region, Bahia, Brazil. Sample data were stratified by reads per sample according to barcode or mini-barcode.
