# Supplementary material for: Invertebrate-Derived DNA (iDNA) to Identify Sand Flies’ Bloodmeal: A Molecular Approach to Identifying Hosts in Blood-Feeding Vectors of Leishmaniasis
Source: Microorganisms. 2025 Nov 21;13(12):2650. doi: 10.3390/microorganisms13122650 (PMC12734529; doi:10.3390/microorganisms13122650)
Supplement: Supplementary file 1 [file microorganisms-13-02650-s001.zip › microorganisms-3934257-supplementary.pdf]

microorganisms

MDPI

Table S1. Vertebrate species identified from sand fly females collected between May 2018 and June 2019 in the Cacao Region, Bahia, Brazil. Sample data were stratified by reads per sample according to barcode or mini-barcode.

Species (Sample)	Barcode or Mini-Barcode	Bloodmeal	Query Cover (%)	Identity (%)	GenBank Accession Number
Nyssomyia whitmani (M3C1PA) *	12S	*Homo sapiens*	100	99.26	FN673842.1
		Dendropsophus elegans	96	100	MK266719.1
		Gallus gallus	99	100	OQ562002.1
	16S	*Mazama* sp.	99	97.7	OP712670.1
		Homo sapiens	100	100	MF621124.1
		Sapajus xanthosternus	100	97.67	NC_021961.1
		Equus caballus	100	100	KT757764.1
		Bos taurus	100	100	KT827217.1
Trichopygomyia longispina (M3C1EC) *	12S	*Dendropsophus elegans*	100	99.21	MK266719.1
		Gracilinanus microtarsus	99	99.25	AJ628389.1
		Homo sapiens	99	100	FN673842.1
		Gallus gallus	99	100	OQ562002.1
	16S	*Homo sapiens*	100	100	MF621124.1
		Mazama sp.	99	97.71	OP712670.1
		Sapajus xanthosternus	100	97.67	NC_021961.1
		Bos taurus	100	100	KT827217.1
		Sus scrofa	100	100	MG250566.1
		Brachyteles arachnoides	99	97.69	MH084743.1
Nyssomyia whitmani (M3C2EA) *	12S	*Homo sapiens*	100	99.26	FN673842.1
		Dendropsophus elegans	100	97.62	MK266719.1
		Gallus gallus	99	100	OQ562002.1
	16S	*Homo sapiens*	100	100	MF621124.1
		Mazama sp.	99	97.71	OP712670.1
		Sapajus xanthosternus	100	97.67	NC_021961.1
		Bos taurus	100	100	KT827217.1
		Sus scrofa	100	100	MG250566.1
Psychodopygus hirsutus (M3C2EB) *	12S	*Dendropsophus elegans*	100	99.21	MK266719.1
		Chiasmocleis schubarti	99	99.21	MH885003.1
	16S	*Homo sapiens*	100	100	MF621124.1
		Mazama sp.	99	97.71	OP712670.1
		Sapajus xanthosternus	100	97.67	NC_021961.1
		Bos taurus	100	100	KT827217.1
		Alouatta guariba	99	100	KY202428.1
		Sus scrofa	100	100	MG250566.1
Nyssomyia whitmani (M2C1I2)	12S	*Homo sapiens*	100	100	NG_009191.3
		Dendropsophus elegans	100	99.21	MK266719.1
	16S	*Homo sapiens*	99	100	CP139522.1
		Sus scrofa	100	100	MG250566.1
		Sapajus xanthosternus	100	97.67	NC_021961.1
		Mazama sp.	99	97.71	OP712670.1
	CytB	*Homo sapiens*	95	99.72	KX697544.1
		Sus scrofa	95	100	DQ512915.1
Trichophoromyia viannamartinsi (M3C2I)	12S	*Homo sapiens*	98	100	FN673842.1
		Gracilinanus microtarsus	100	99.26	AJ628389.1
		Canis lupus	100	100	OM743430.1

	16S	*Homo sapiens*	100	100	MF621124.1
		Mazama sp.	99	97.71	OP712670.1
		Sus scrofa	100	100	MG250566.1
		Bos taurus	100	100	KT827217.1
		Sapajus xanthosternus	100	97.67	NC_021961.1
		Canis lupus	99	100	LR742783.1
	CytB	*Homo sapiens*	95	100	KX697544.1
		Gallus gallus	95	100	DQ512917.1
		Canis lupus	94	99.72	DQ309764.1
Trichophoromyia viannamartinsi (M4C2E)	12S	*Equus asinus*	98	100	MK982180.1
		Gallus gallus	100	100	MW524218.1
		Phylomedusa bahiana	100	99.21	NC_067554.1
		Homo sapiens	100	98.44	NG_009191.3
		Equus caballus	98	97.04	KT221831.1
	16S	*Equus asinus*	100	100	PP049800.1
		Mazama sp.	99	97.71	OP712670.1
		Homo sapiens	100	100	MF621124.1
		Sapajus xanthosternus	100	97.67	NC_021961.1
		Brachyteles arachnoides	99	97.69	MH084743.1
		Equus caballus	100	98.46	MZ040622.1
		Sus scrofa	100	100	MG250566.1
		Bos taurus	100	99.24	KT827217.1
	CytB	*Gallus gallus*	97	100	AJ401080.1
		Homo sapiens	96	100	KX697544.1
		Canis lupus	97	100	DQ309764.1
Migonemyia migonei (M5C1E)	12S	*Coendou prehensilis*	100	94.74	KX381447.1
		Cuniculus paca	97	93.8	KX381423.1
	16S	*Cuniculus paca*	100	91.04	NC_079967.1
		Mazama sp.	99	97.71	OP712670.1
		Homo sapiens	100	100	MF621124.1
		Sapajus xanthosternus	100	97.67	NC_021961.1
	CytB	*Homo sapiens*	95	100	KX697544.1
		Canis lupus	94	99.72	DQ309764.1
		Chaetomys subspinosus	94	97.76	EU544660.1
Migonemyia migonei (M6C2E)	12S	*Equus asinus*	98	100	MK982180.1
		Gallus gallus	100	100	MW524218.1
		Homo sapiens	99	99.26	FN673842.1
	16S	*Mazama* sp.	99	97.71	OP712670.1
		Equus asinus	100	100	PP049800.1
		Homo sapiens	100	100	MF621124.1
		Bos taurus	100	100	KT827217.1
		Sapajus xanthosternus	100	97.67	NC_021961.1
		Sus scrofa	100	100	MG250566.1
		Brachyteles arachnoides	99	97.69	MH084743.1
		Equus caballus	100	98.46	MZ040622.1
	CytB	*Homo sapiens*	95	100	KX697544.1
		Equus caballus	95	98.88	AY819737.1
		Equus asinus	94	98.6	MK982180.1
		Gallus gallus	95	100	DQ512917.1
		Canis lupus	94	100	DQ309764.1
		Sus scrofa	95	100	DQ512915.1
		Chaetomys subspinosus	94	94.68	EU544660.1
Migonemyia migonei	12S	*Gallus gallus*	99	100	OQ562002.1

(M7C2P1)		*Chiasmocleis schubarti*	99	99.21	MH885003.1
		Homo sapiens	99	98.52	CP139520.1
	16S	*Mazama* sp.	99	97.71	OP712670.1
		Homo sapiens	100	100	MF621124.1
		Sapajus xanthosternus	100	97.67	NC_021961.1
		Brachyteles arachnoides	99	97.69	MH084743.1
		Sus scrofa	100	100	MG250566.1
		Bos taurus	100	100	KT827217.1
		Canis lupus	99	100	LR742783.1
Trichophoromyia viannamartinsi (M3C1PB) *	CytB	*Homo sapiens*	95	100	KX697544.1
		Eunectes murinus	94	97.74	U69809.1
		Chaetomys subspinosus	94	97.76	EU544660.1
		Nyssomyia sp.	94	92.09	NC_026898.1
		Equus asinus	94	98.31	MK982180.1
Pintomyia fischeri (M6C3P)	CytB	*Homo sapiens*	95	100	KX697544.1
		Gallus gallus	95	100	DQ512917.1
		Sus scrofa	95	100	DQ512915.1
		Chaetomys subspinosus	94	97.48	EU544660.1
Evandromyia bahiensis (M9C2E)	CytB	*Homo sapiens*	95	100	KX697544.1
		Gallus gallus	95	100	DQ512917.1
		Trinomys albispinus	95	98.61	U34856.1

* Sample with at least one female non-engorged phlebotomine sand fly infected by *Leishmania* (*Vi-annia*) *braziliensis*.
